# Control of breathing

**DOI:** 10.1186/1824-7288-40-S2-A33

**Published:** 2014-10-09

**Authors:** Corrado Moretti, Stefano Luciani, Rosanna Grossi, Caterina S Barbàra, Paola Papoff

**Affiliations:** 1 Department of Paediatrics, Paediatric Emergency and Intensive Care, Sapienza University of Rome, Rome, Italy

## 

Late preterm infants have been called “great imposters” [1] because they often appear to be and are therefore treated as term infants, however their brainstem development and neural control of respiration are less mature than in full-term infants. During late gestation there are dramatic developmental changes in the brainstem and forebrain: at 34 gestational weeks the total brain weight is 65% of that at term [2]. The control of lung volume, laryngeal reflexes, upper airways, chemoreceptor activity, coordination of sucking, swallowing, breathing, the incidence of apnea and periodic breathing and the control of heart rate are still developing during late gestation and the degree of maturity of these mechanisms appears to lie on a continuum that extends from more immature infants to after term. Also, these reflexes are influenced by sleep-wake states whose neurobiological development is still evolving. The brainstem is not completely mature even at term, as confirmed by the fact that its myelination, the marker of completed neuronal/axonal maturation, is still incomplete at that time. This protracted maturation is not surprising, considering that the neurodevelopment of the respiratory control and sleep-wake cycle continues into infancy. The few data on control of breathing in late preterm infants indicate that ventilatory responses to CO_2_ and hypoxia as well as autonomic control of heart rate are not yet mature at 36 weeks PMA. Clinically, late preterm infants have more apnea and periodic breathing than term infants and immature coordination of sucking, swallowing and breathing often delay their ability to feed without episodes of bradycardia, desaturation and even apnea [3]. The incidence of apparent life-threatening events is more common in preterm infants (8-10%) than full-term infants (1% or less). In the Collaborative Home Infant Monitoring Evaluation studies the frequency of conventional and extreme (clinically relevant) events in near term infants is intermediate between preterm infants <34 weeks at birth and full-term infants [4].

Clinical data indicate that late preterm are also predisposed to wheezing in infancy and early childhood although not exposed to excessive supplemental oxygen or ventilator support [5]. Exposure to 21% oxygen could represent premature exposure to hyperoxic environment compared to that in utero, with long-term effects on the still immature conducting airways. Recent data demonstrate that immature human airway smooth muscle are highly sensitive to even short durations of hyperoxia, with increased proliferation at moderate levels of oxygen (< 60%) but apoptosis at higher levels [6].
